# Endothelial cell–derived exosomal circHIPK3 promotes the proliferation of vascular smooth muscle cells induced by high glucose via the miR-106a-5p/Foxo1/Vcam1 pathway

**DOI:** 10.18632/aging.203742

**Published:** 2021-12-10

**Authors:** Shaohua Wang, Min Shi, Jiao Li, Yuanyuan Zhang, Wenjing Wang, Peixin Xu, Yongjun Li

**Affiliations:** 1 Department of Clinical Laboratory, Hebei Key Laboratory of Laboratory Medicine, The Second Hospital of Hebei Medical University, Shijiazhuang, Hebei 050000, P.R. China

**Keywords:** circHIPK3, exosomes, cell communication, VSMCs, high glucose

## Abstract

The abnormal proliferation of vascular smooth muscle cells (VSMCs) plays an important role in the development and progression of diabetic vascular complications. In high-glucose (HG) conditions, endothelial cells (ECs) act as the first barrier to damaging stimuli and trigger a multi-response, including EC and VSMC crosstalk. However, the crosstalk pathways between ECs and VSMCs under HG conditions remain unclear. This study aimed to explore the roles and underlying mechanism of exosomes derived from ECs in the crosstalk between ECs and VSMCs. Our results showed that mouse aortic endothelial cell (MAEC)–secreted exosomes could promote the proliferation and inhibit the apoptosis of VSMCs induced by HG. Furthermore, we isolated the exosomes secreted by MAECs and found that exosomes derived from MAECs that were exposed to HG could transfer circHIPK3, which is enriched in MAEC-derived exosomes, to VSMCs. Exosomal circHIPK3 promoted the proliferation and inhibited the apoptosis of VSMCs. circHIPK3 sponged miR-106a-5p to relieve its repression of forkhead box O1 (Foxo1) expression. The increased expression of Foxo1 acted as a transcription factor to promote Vcam1 expression, thus facilitating the uptake of MAEC-derived exosomes by VSMCs. The results of this study suggested that exosomal circHIPK3 derived from MAECs promotes the proliferation of VSMCs induced by HG via the miR-106a-5p/Foxo1/Vcam1 pathway.

## INTRODUCTION

Vascular complications are the major causes related to morbidity and mortality in patients with type 2 diabetes mellitus. Considering that continuous high-glucose (HG) conditions can lead to vascular dysfunction, multiple types of cells are involved in this process, such as endothelial cells (ECs), vascular smooth muscle cells (VSMCs), and macrophages [1]. ECs are the first barrier exposed to various damaging stimuli (such as HG) and can alter the functions of other cells in different ways. For example, glucose at high levels can penetrate ECs by passive diffusion, which induces endothelial dysfunction via oxidative stress, inflammation, and apoptosis [2]. Endothelial dysfunction can lead to VSMC activation, which induces altered proliferation and migration [3]. Therefore, the crosstalk and interaction between ECs and VSMCs play an essential part in regulating diabetic vascular disease progression; however, the underlying mechanisms of this interaction have not been comprehensively studied and remain unclear.

Exosomes are a subset of extracellular vesicles, 40–150 nm in diameter, and secreted by most cells [4]. Recent studies have revealed the important roles of exosomes in cellular communication, and these roles are related to various physiological and pathological processes, including cardiovascular disease [5]. For instance, the exosomes secreted by smooth muscle cells carry miR-155, which induces endothelial injury and enhances atherosclerotic progression [6]. Moreover, the exosomes secreted by nicotine-stimulated macrophages can accelerate the progression of atherosclerosis by increasing the migration and proliferation of VSMCs [7]. Exosomes perform their functions depending on their carrying proteins, lipids, and RNAs [8]. Circular RNAs (circRNAs) are non-coding RNAs that are widely involved in various processes, such as cell proliferation, differentiation, and apoptosis [9]. CircRNAs possess the closed covalent loop structure, which is known for its high stability in physiological fluids. Furthermore, exosomes can carry circRNAs to a recipient cell to induce various functions [10]. For example, the exosomal circEhmt1 released by hypoxia-pretreated pericytes is related to HG-induced microvascular dysfunction [11]. In pancreatic cancer, tumor-released exosomal circRNA PDE8A can promote growth and invasion via the miR-338/MACC1/MET axis [12]. circHIPK3 is composed of the second exon from *HIPK3*, which is involved in regulating apoptosis, proliferation, migration, and angiogenesis. In diabetes mellitus, circHIPK3 alleviated retinal vascular dysfunction [13]. Exosomal circHIPK3 released from hypoxia-pretreated cardiomyocytes suppresses oxidative stress–induced cardiac microvascular EC dysfunction [14]. However, the functions and underlying mechanism related to the mediation of EC–VSMC communication by circHIPK3 in HG-induced vascular complications have not been comprehensively studied and are not yet understood.

In this study, we detected the roles and underlying mechanism of mouse aortic endothelial cell (MAEC)–derived exosomal circHIPK3 on the phenotype of VSMCs in HG conditions. This study revealed that exosomal circHIPK3 derived from MAECs promotes the proliferation of VSMCs induced by HG via the miR-106a-5p/Foxo1/Vcam1 axis.

## RESULTS

### HG promoted exosome secretion in MAECs

**Figure 1:**
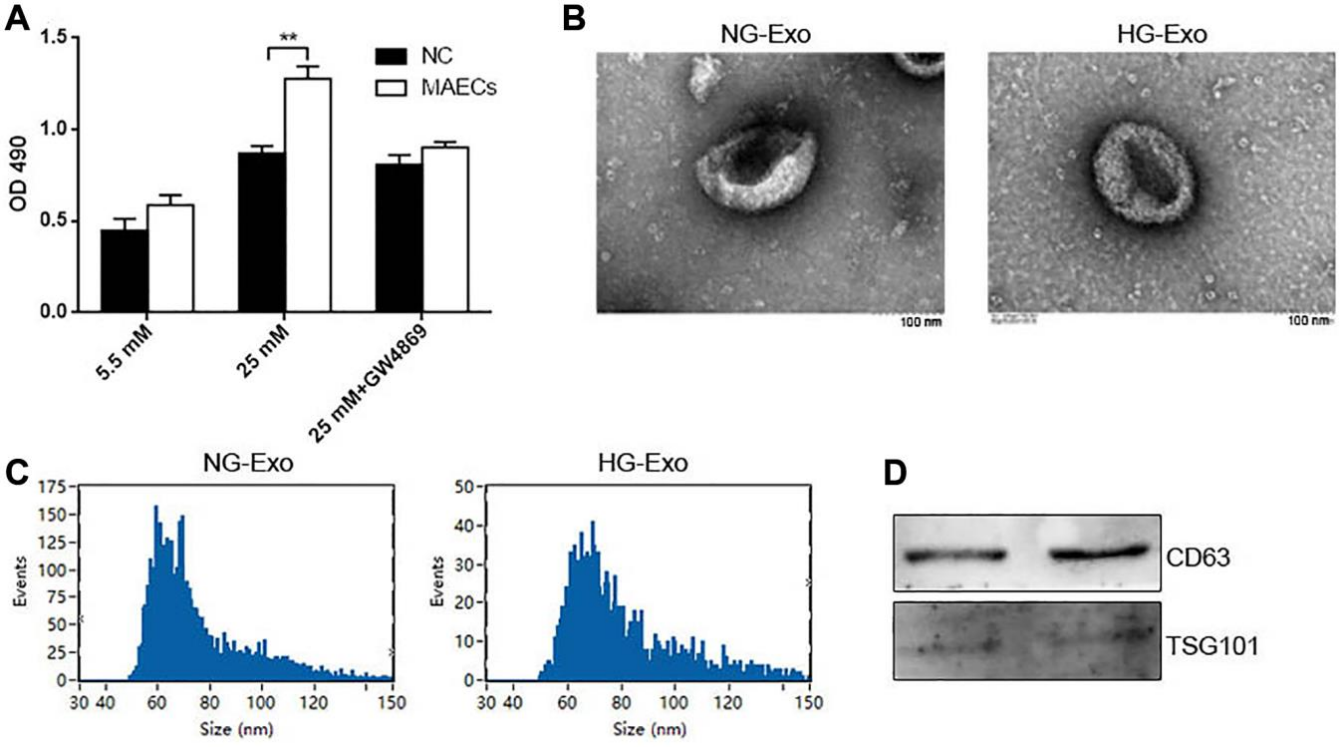
**HG promotes exosomes secretion in MAECs.** (**A**) CCK-8 was used to detect cell viability in VSMCs co-cultured with MAECs (^**^p < 0.01 MAECs vs. NC). (**B**) Cell viability was detected by Edu assay. (**C**) NTA measured the size distribution of exosomes from an HG or NG condition. (**D**) Western blot analysis detected the expression of exosome markers CD63 and TSG 101.

To understand whether crosstalk occurs between ECs and VSMCs under an HG condition, we co-cultured MAECs and VSMCs in normal glucose (NG; 5.5 mM) or HG (25 mM) with a transwell system. MAECs were loaded on top of a transwell that transferred cellular secretions but prevented cell transfer. We observed that in an HG condition, MAECs could promote the proliferation of VSMCs, as shown by the CCK-8 assay (Figure 1A). Once GW4869 (exosome inhibitor) was added, it abolished this proliferation at a large scale (Figure 1A). These results indicated that exosomes might be involved in the proliferation of VSMCs due to ECs under HG conditions. To further confirm this perspective, we isolated and characterized exosomes from an HG or NG culture medium of MAECs. Transmission electron microscopy (TEM) showed a typical cup-shaped morphology with a size of 40–150 nm (Figure 1B). Furthermore, after using the nanoparticle tracking analysis (NTA) assay, we did not observe any differences related to the concentration of exosomes from HG (HG-Exo) and NG (NG-Exo) conditions, but the size peak of the exosomes was at 59.25 nm for NG-Exo and 69.25 nm for HG-Exo (Figure 1C). Western blot analysis showed the presence of exosome surface markers, including CD63 and TSG101 (Figure 1D).

### Effects of MAEC-derived exosomes on VSMC proliferation and apoptosis

**Figure 2:**
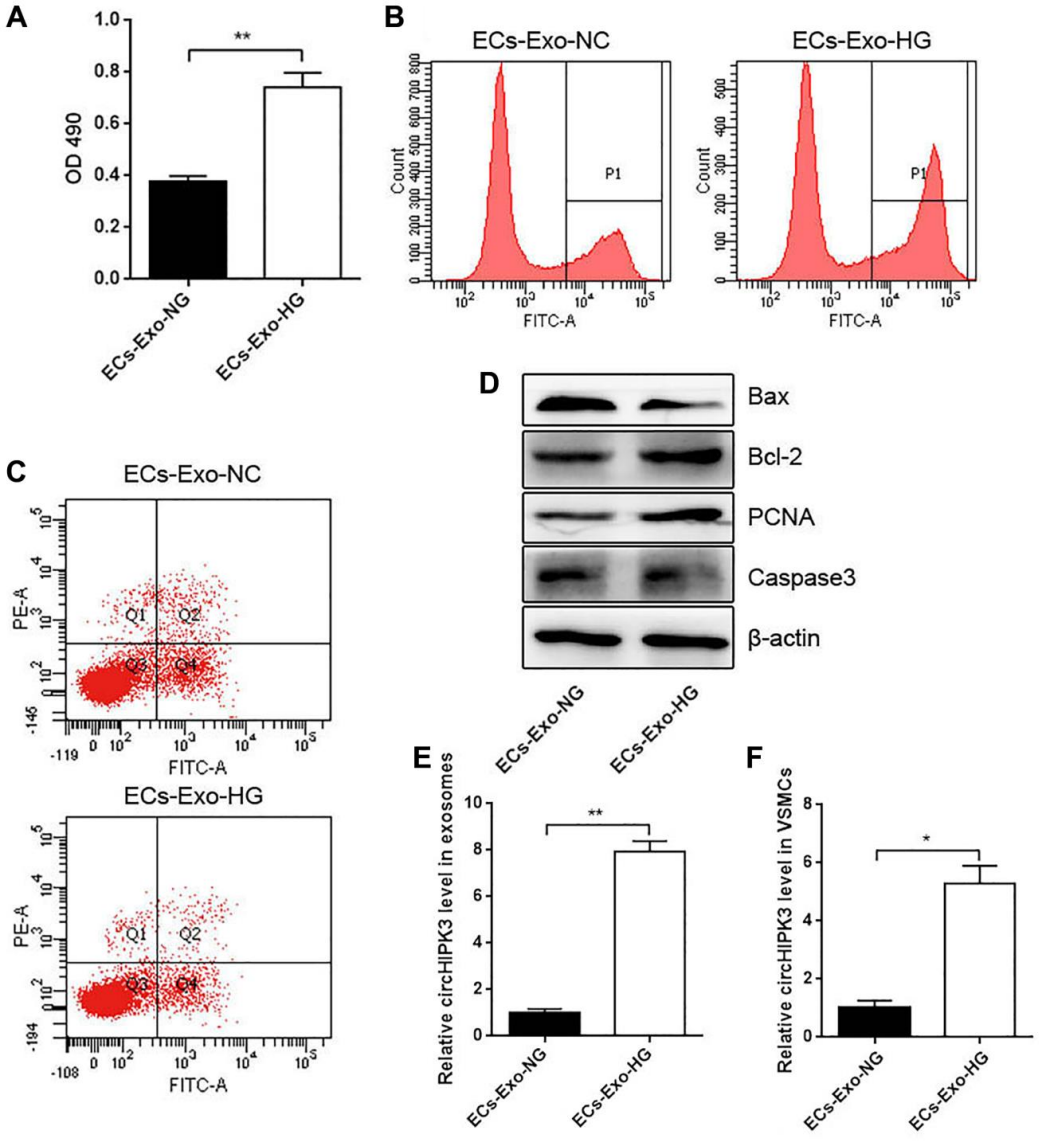
**Effects of MAEC-derived exosomes on VSMC proliferation and apoptosis.** (**A**) CCK-8 was used to detect cell viability in VSMCs incubated with exosomes isolated from MAECs cultured in an HG or NG medium (^**^p < 0.01 ECs-Exo-NG vs. ECs-Exo-HG). (**B**) Edu assay was used to detect cell viability by FCM. (**C**) FCM detected the apoptosis of VSMCs. (**D**) Western blot analysis detected the expression of Bax, Bcl2, PCNA, and Caspase 3. (**E**) The relative expression of circHIPK3 in the exosomes of ECs-Exo-NG or ECs-Exo-HG detected by qRT-PCR (^**^p < 0.01 ECs-Exo-NG vs. ECs-Exo-HG). (**F**) The relative expression of circHIPK3 in VSMCs incubated with ECs-Exo-NG or ECs-Exo-HG detected by qRT-PCR (^*^p < 0.05 ECs-Exo-NG vs. ECs-Exo-HG).

To understand the crosstalk between MAECs and VSMCs in HG conditions, we incubated VSMCs with exosomes isolated from a normal MAEC medium (ECs-Exo-NG; 5.5 mM) or an HG medium (ECs-Exo-HG; 25 mM) for 24 h and performed CCK-8 and 5-ethynyl-2' -deoxyuridine (EdU) assays. VSMCs incubated with ECs-Exo-HG showed markedly increased viability compared with ECs-Exo-NG (Figure 2A and 2B). Flow cytometry (FCM) analysis showed that VSMCs incubated with ECs-Exo-HG exhibited decreased apoptosis compared with ECs-Exo-NG after inducing apoptosis by H_2_O_2_ (Figure 2C). Western blot analysis showed that the expressions of Proliferating Cell Nuclear Antigen (PCNA) and Bcl2 were increased and those of Caspase 3 and Bax were decreased in VSMCs incubated with ECs-Exo-HG compared with those incubated with ECs-Exo-NG (Figure 2D). These results indicated that exosomes derived from ECs promoted proliferation and inhibited apoptosis in VSMCs. To explore the underlying molecular mechanism of this phenomenon, we also isolated exosomes from the MAEC culture medium and found that the level of circHIPK3 increased in the ECs-Exo-HG group compared with the ECs-Exo-NG group (Figure 2E). In addition, the level of circHIPK3 increased significantly in VSMCs incubated with ECs-Exo-HG compared with those incubated with ECs-Exo-NG (Figure 2F).

### MAEC-derived exosomal circHIPK3 promoted the proliferation and inhibited the apoptosis of VSMCs

**Figure 3:**
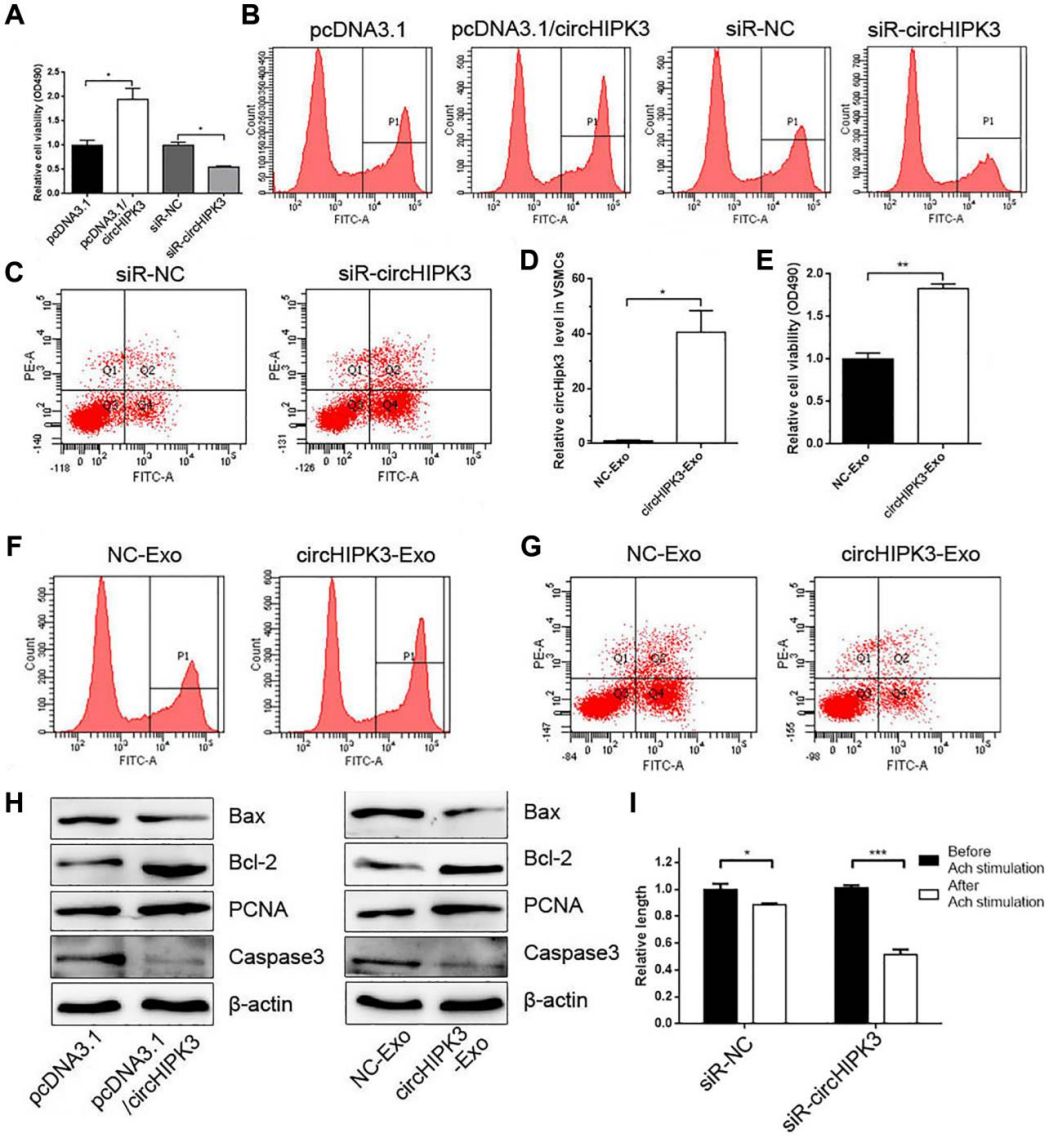
**MAEC-derived exosomal circHIPK3 promotes VSMC proliferation and inhibited VSMC apoptosis.** (**A**) CCK-8 was used to detect cell viability in VSMCs when overexpressing or knocking down circHIPK3 in VSMCs (^*^p < 0.05 pcDNA3.1/circHIPK3 vs. pcDNA3.1, ^*^p < 0.05 siR-circHIPK3 vs. siR-NC). (**B**) Edu assay was used to detect cell viability by FCM. (**C**) FCM detected the apoptosis of VSMCs transfected with siR-circHIPK3 or siR-NC. (**D**) The relative expression of circHIPK3 in VSMCs incubated with exosomes isolated from 293A cells overexpressing circHIPK3 (^*^p < 0.05 NC-Exo vs. circHIPK3-Exo). (**E**) CCK-8 was used to detect cell viability in VSMCs incubated with NC-Exo or circHIPK3-Exo (^**^p < 0.01 NC-Exo vs. circHIPK3-Exo). (**F**) Edu assay was used to detect cell viability by FCM. (**G**) FCM detected the apoptosis of VSMCs. (**H**) Western blot analysis detected the expression of Bax, Bcl2, PCNA, and Caspase 3. (**I**) Cell length was calculated after infection with siR-NC or siR-circHIPK3 before and after inducing by Ach (^*^p < 0.05 Before Ach stimulation vs. After Ach stimulation for siR-NC, ^***^p < 0.001 Before Ach stimulation vs. After Ach stimulation for siR-circHIPK3).

Given that exosomes derived from ECs change the phenotype of VSMCs and alter the expression of circHIPK3 in VSMCs, we focused on exosomal circHIPK3 derived from ECs on the phenotype of VSMCs. CCK-8 and EdU assays were performed after overexpressing or knocking down circHIPK3. The results showed that circHIPK3 overexpression and knockdown promoted and inhibited the cell viability of VSMCs, respectively (Figure 3A and 3B). FCM analysis showed that siR-circHIPK3 could promote the apoptosis of VSMCs (Figure 3C). We also transfected pcDNA3.1/circHIPK3 (circHIPK3-Exo) or pcDNA3.1 (NC-Exo) in 293A cells and isolated exosomes to incubate VSMCs. The Real-Time Quantitative Reverse Transcription PCR (qRT-PCR) results showed that the circHIPK3 level increased in the circHIPK3-Exo group compared with that in the NC-Exo group in VSMCs (Figure 3D). CCK-8 and EdU assays showed that the cell viability of VSMCs increased in the circHIPK3-Exo group compared with that in the NC-Exo group (Figure 3E and 3F). By contrast, FCM showed decreased apoptosis in VSMCs incubated with circHIPK3-Exo compared with NC-Exo after inducing apoptosis by H_2_O_2_ (Figure 3G). Nevertheless, western blot analysis showed that the expressions of PCNA and Bcl2 increased in VSMCs transfected with pcDNA3.1/circHIPK3 or incubated with circHIPK3-Exo compared with those of the control group. The expression of Bax and Caspase 3 decreased in the pcDNA3.1/circHIPK3 group and circHIPK3-Exo group (Figure 3H). Considering that VSMCs regulate vessel tone and blood pressure by contraction and relaxation, we examined the effect of circHIPK3 on VSMC contraction induced by acetylcholine (Ach). The results showed that circHIPK3 knockdown promoted Ach-induced VSMC contraction (Figure 3I).

### circHIPK3 promoted Foxo1 expression via sponging miR-106a-5p

**Figure 4:**
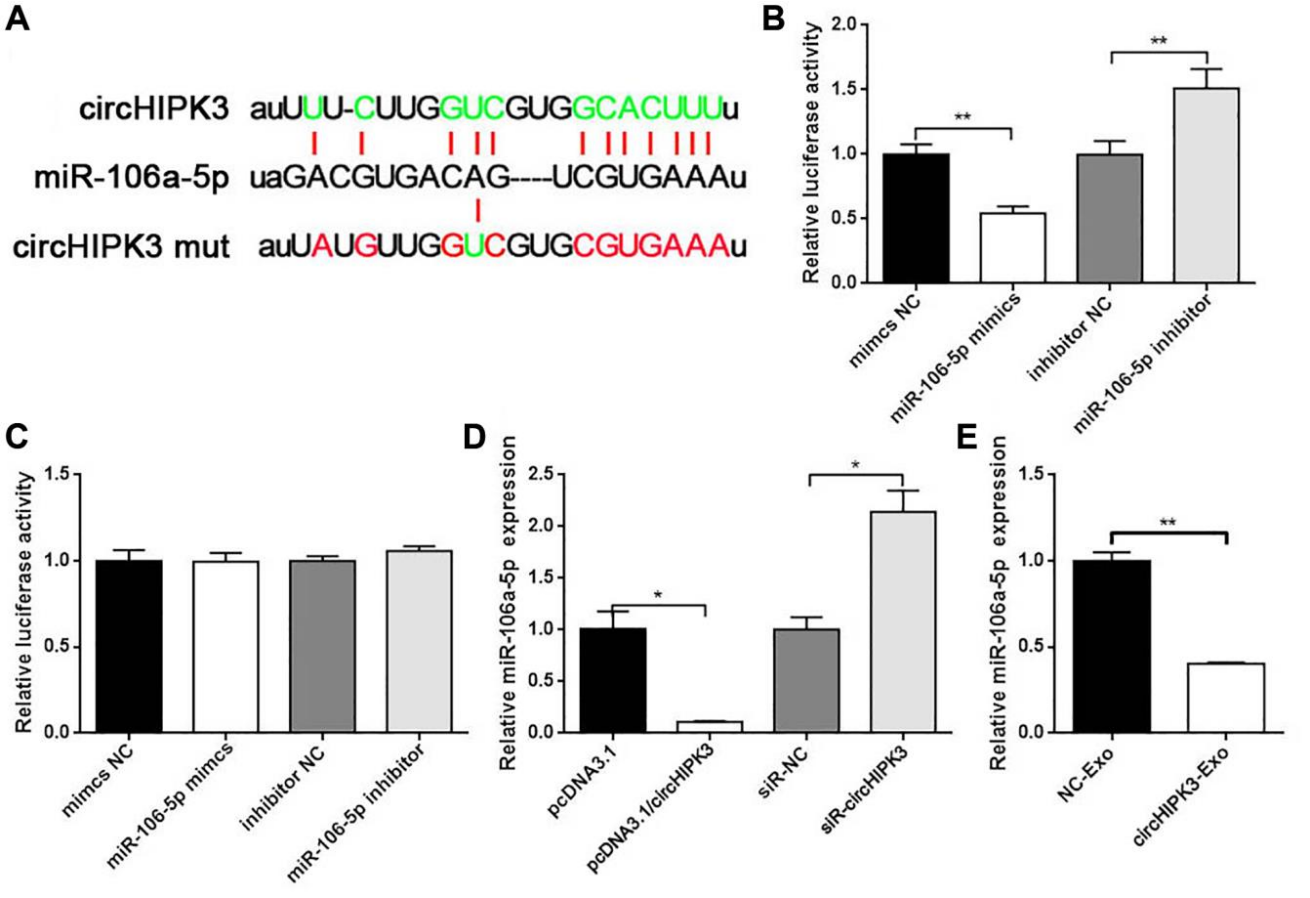
**circHIPK3 sponges miR-106a-5p.** (**A**) Putative miR-106a-5p binding sequences in wild-type or mutated circHIPK3. (**B**) Luciferase report assay showed the direct relationship between wild-type circHIPK3 and miR-106a-5p (^**^p < 0.01 mimics NC vs. miR-106-5p, ^**^p < 0.01 inhibitor NC vs. miR-106-5p inhibitor). (**C**) Luciferase report assay showed the direct relationship between circHIPK3 mutation and miR-106a-5p. (**D**) qRT-PCR was performed to detect the amount of miR-106a-5p mRNA in VSMCs after the overexpression or knockdown of circHIPK3 (^*^p < 0.05 pcDNA3.1/circHIPK3 vs. pcDNA3.1, ^*^p < 0.05 siR-circHIPK3 vs. siR-NC). (**E**) miR-106a-5p level was detected by qRT-PCR when cells were incubated with exosomes (^**^p < 0.01 NC-Exo vs. circHIPK3-Exo).

To further evaluate the underlying molecular mechanism of circHIPK3 on the VSMC phenotype, we used StarBase to predict the binding sites of miRNAs in the whole circHIPK3 sequence. We found that circHIPK3 contained sequences complementary to miR-106a (Figure 4A). We constructed luciferase plasmid (pmirGLO/circHIPK3 wt) and co-transfected pmirGLO/circHIPK3 wt with miR-106a-5p mimics or miR-106a-5p inhibitor into 293A cells. The results showed that when pmirGLO/circHIPK3 was co-transfected with miR-106a-5p mimics, the luciferase activity decreased compared with that of the control group. Once pmirGLO/circHIPK3 was co-transfected with miR-106a-5p inhibitor, the luciferase activity increased (Figure 4B). Moreover, we also constructed the mutation plasmid (pmirGLO/circHIPK3 mut) that contained the mutation sites of the binding sites between them. No effects on luciferase activity were observed when cells were co-transfected with pmirGLO/circHIPK3 mut along with miR-106a-5p mimics or miR-106a-5p inhibitor (Figure 4C). qRT-PCR was performed to evaluate the regulation between circHIPK3 and miR-106a-5p. Our results showed that circHIPK3 led to a decrease in miR-106a-5p expression (Figure 4D). CircHIPK3-Exo inhibited miR-106a-5p expression compared to NC-Exo (Figure 4E). These results revealed that exosomal circHIPK3 could bind miR-106a-5p and regulate its expression *in vitro*.

**Figure 5:**
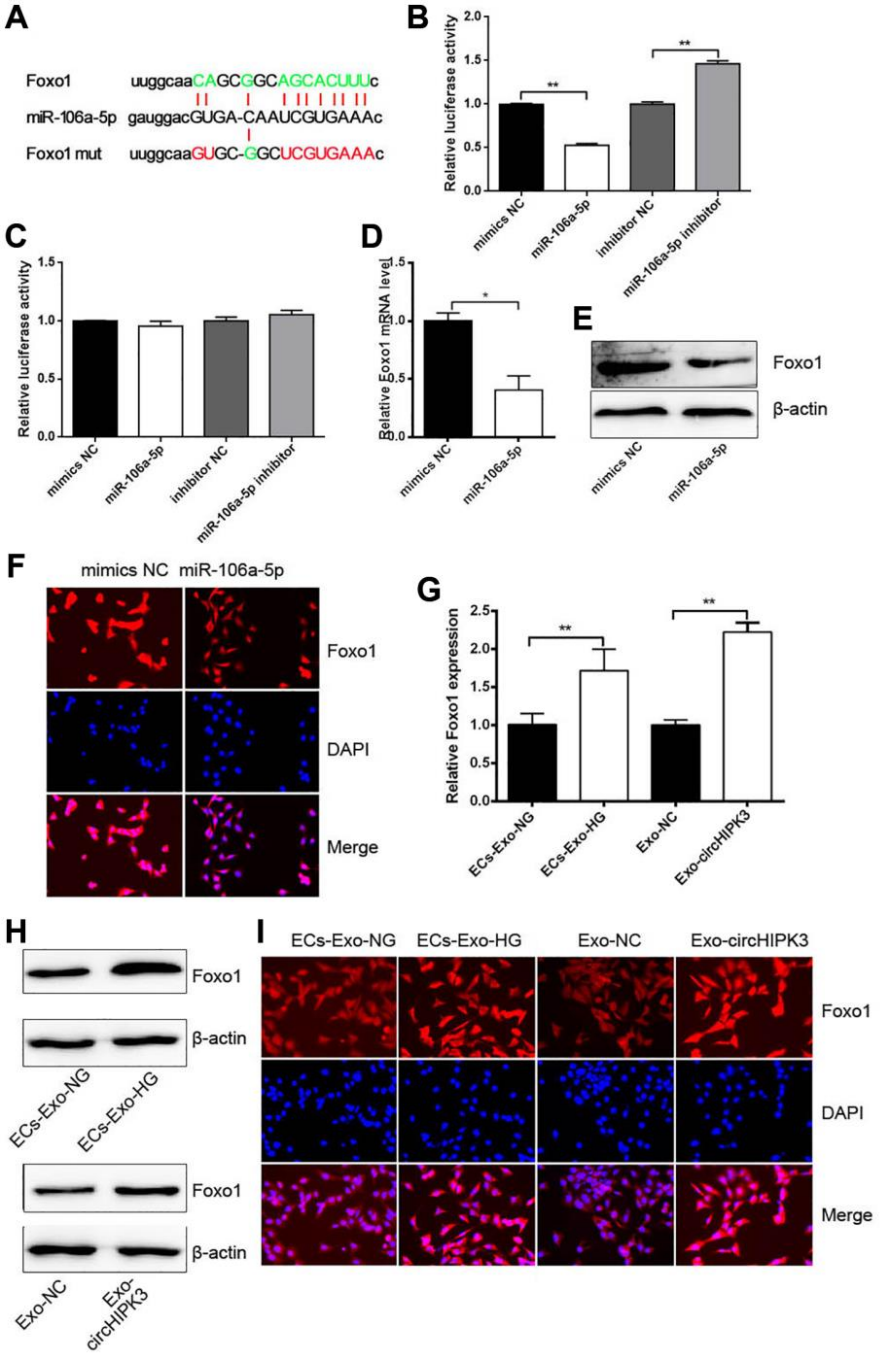
**miR-106a-5p inhibits Foxo1 expression by targeting its 3’UTR.** (**A**) Putative miR-106a-5p binding sequences in wild-type or mutated Foxo1 3’UTR. (**B**) Luciferase report assay showed the direct relationship between wild-type Foxo1 3’UTR and miR-106a-5p (^**^p < 0.01 miR-106a-5p vs. mimics NC, ^**^p < 0.01 miR-106a-5p inhibitor vs. inhibitor NC). (**C**) Luciferase report assay showed the direct relationship between Foxo1 3’UTR mutation and miR-106a-5p. (**D**) qRT-PCR, (**E**) western blot analysis, and (**F**) IF staining detected Foxo1 mRNA and protein levels in VSMCs overexpressing miR-106a-5p (^*^p < 0.05 miR-106a-5p vs. mimics NC). (**G**) qRT-PCR, (**H**) western blot analysis, and (**I**) IF staining detected Foxo1 mRNA and protein levels in VSMCs incubated with exosomes (^**^p < 0.01 ECs-Exo-NG vs. ECs-Exo-HG, ^**^p < 0.01 NC-Exo vs. circHIPK3-Exo).

Given that miRNAs regulate mRNA expression by binding to the 3′UTR section of mRNAs, we predicted that Foxo1 3′UTR had the binding sites of miR-106a-5p (Figure 5A). Dual luciferase assay was performed, and the results showed that luciferase activity was reduced when 293A cells were co-transfected with miR-106a-5p mimics and pmirGLO/Foxo1 3′UTR wt compared with when 293A cells were co-transfected with mimics NC and pmirGLO/Foxo1 3′UTR wt. Luciferase activity was increased when cells were co-transfected with miR-106a-5p inhibitor and pmirGLO/Foxo1 3′UTR wt (Figure 5B). No changes were observed when 293A cells were co-transfected with miR-106a-5p mimics or miR-106a-5p inhibitor and pmirGLO/Foxo1 3′UTR mut compared with their corresponding control group (Figure 5C). qRT-PCR, western blot analysis, and Immunofluorescence (IF) revealed that the overexpression of miR-106a-5p decreased Foxo1 mRNA and protein levels (Figure 5D–5F). Nevertheless, VSMCs incubated with ECs-Exo-HG or Exo-circHIPK3 promoted Foxo1 mRNA and protein expressions compared with their corresponding control (Figure 5G–5I). These results indicated that circHIPK3 promoted Foxo1 expression via sponging miR-106a-5p in VSMCs.

### miR-106a-5p overexpression and Foxo1 knockdown inhibited proliferation and promoted apoptosis in VSMCs

**Figure 6:**
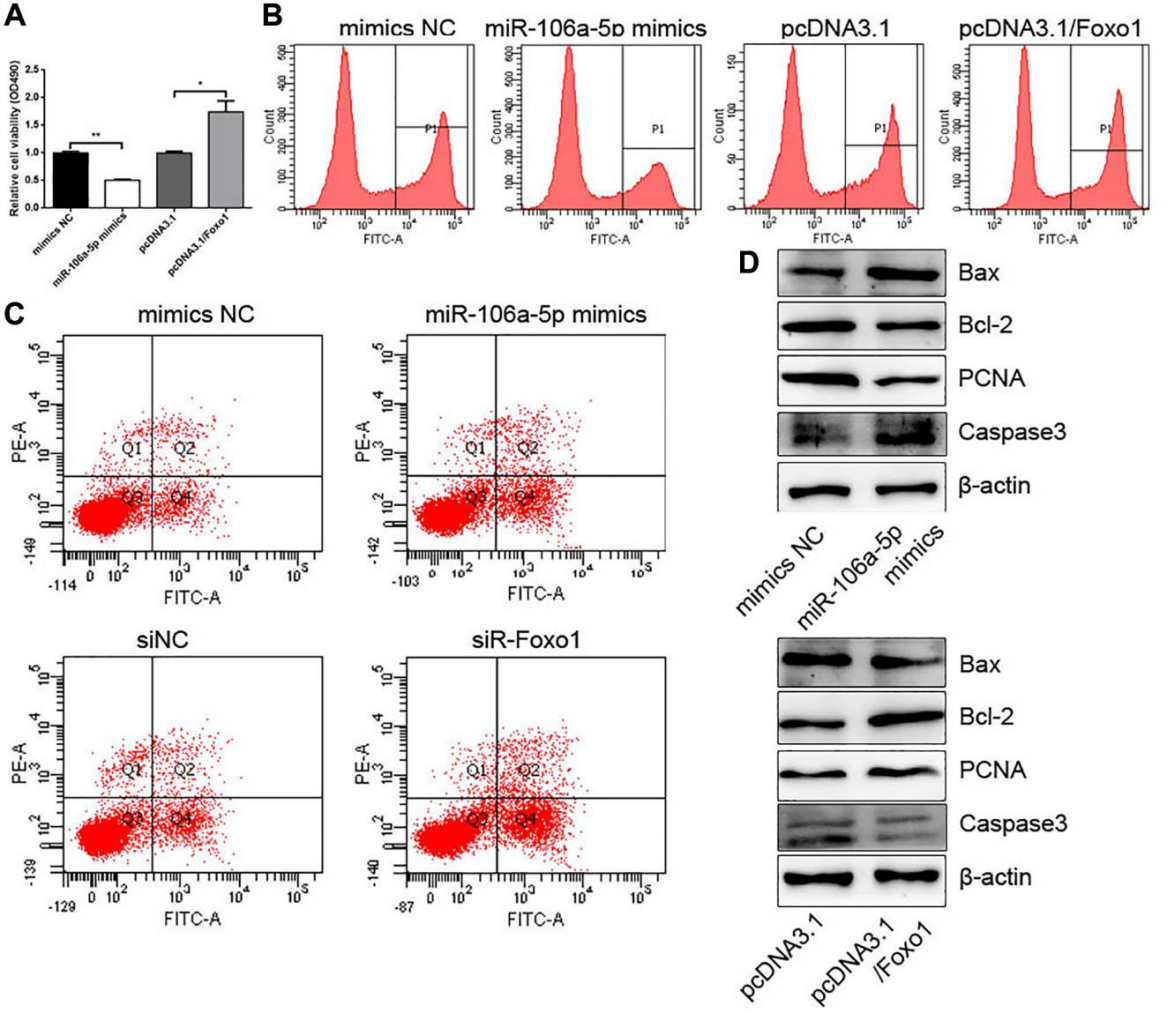
**miR-106a-5p overexpression and Foxo1 knockdown inhibits the proliferation and promotes the apoptosis of VSMCs.** (**A**) CCK-8 was used to detect cell viability in VSMCs overexpressing miR-106a-5p or Foxo1 (^**^p < 0.01 miR-106a-5p vs. mimics NC, ^*^p < 0.05 pcDNA3.1/Foxo1 vs. pcDNA3.1). (**B**) Edu assay was used to detect cell viability by FCM. (**C**) FCM detected the apoptosis of VSMCs. (**D**) Western blot analysis detected the expression of Bax, Bcl2, PCNA, and Caspase 3.

We also confirmed the roles of miR-106a-5p and Foxo1 in the proliferation and apoptosis of VSMCs. CCK-8 and EdU assays showed that miR-106-5p decreased VSMC proliferation, whereas Foxo1 promoted VSMC proliferation (Figure 6A and 6B). FCM analysis showed that miR-106-5p overexpression and Foxo1 knockdown increased the apoptosis of VSMCs (Figure 6C). Western blot analysis showed that miR-106-5p overexpression could increase Caspase 3 and Bax expressions and decrease PCNA and Bcl2 expressions, whereas Foxo1 overexpression exhibited the opposite effects (Figure 6D).

### Foxo1 served as a transcription factor to regulate Vcam1 expression

**Figure 7:**
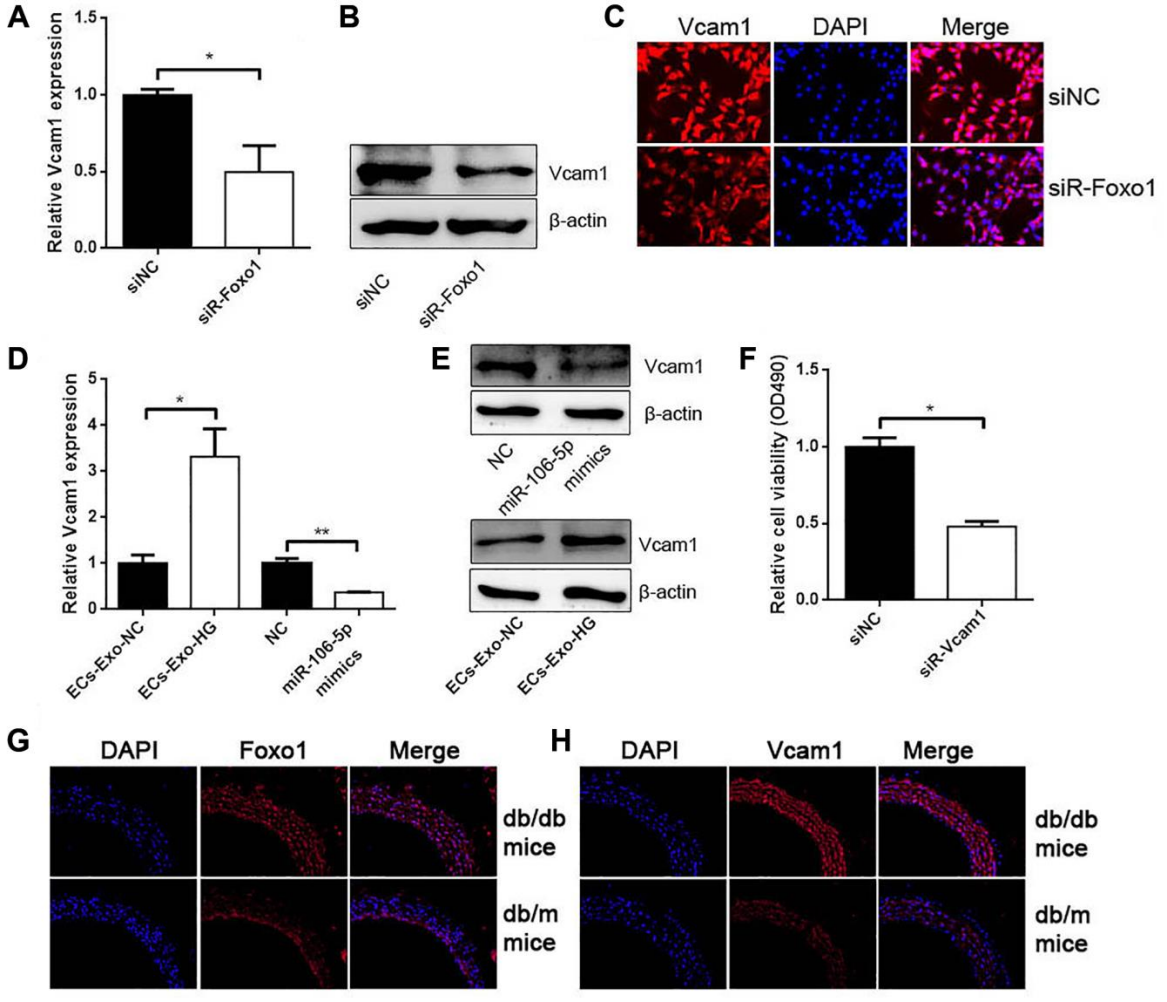
**Foxo1 serves as a transcription factor to regulate Vcam1 expression.** (**A**) qRT-PCR, (**B**) western blot analysis, and (**C**) IF staining detected Vcam1 mRNA and protein levels in VSMCs knocking down Foxo1 (^*^p < 0.05 siR-Foxo1 vs. siNC). (**D**) qRT-PCR and (**E**) western blot analysis detected Vcam1 mRNA and protein levels in VSMCs (^*^p < 0.05 ECs-Exo-HG vs. ECs-Exo-NG, ^**^p < 0.01 miR-106a-5p vs. NC). (**F**) CCK-8 was used to detect cell viability in VSMCs knocking down Vcam1 (^*^p < 0.05 siR-Vcam1 vs. siNC). (**G**–**H**) The expression of Foxo1 (**G**) and Vcam1 (**H**) in the aorta of db/db mice and db/m mice by IF.

Previous studies revealed that Foxo1 could promote Vcam1 expression as a transcription factor. The results obtained from qRT-PCR, western blot analysis, and IF found that Foxo1 knockdown inhibited Vcam1 expression (Figure 7A–7C). ECs-Exo-HG promoted Vcam1 mRNA and protein levels compared with their negative control (Figure 7D and 7E). miR-106a-5p overexpression inhibited Vcam1 expression (Figure 7D and 7E). CCK-8 assay showed that Vcam1 knockdown could inhibit VSMC proliferation (Figure 7F). In addition, we used db/db mice and the control db/m mice. As expected, IF for Foxo1 and Vcam1 revealed that Foxo1 and Vcam1 expressions were significantly increased in the aorta of db/db mice compared with the control db/m mice (Figure 7G and 7H).

### Vcam1 mediated the VSMC uptake of MAEC-derived exosomes

**Figure 8:**
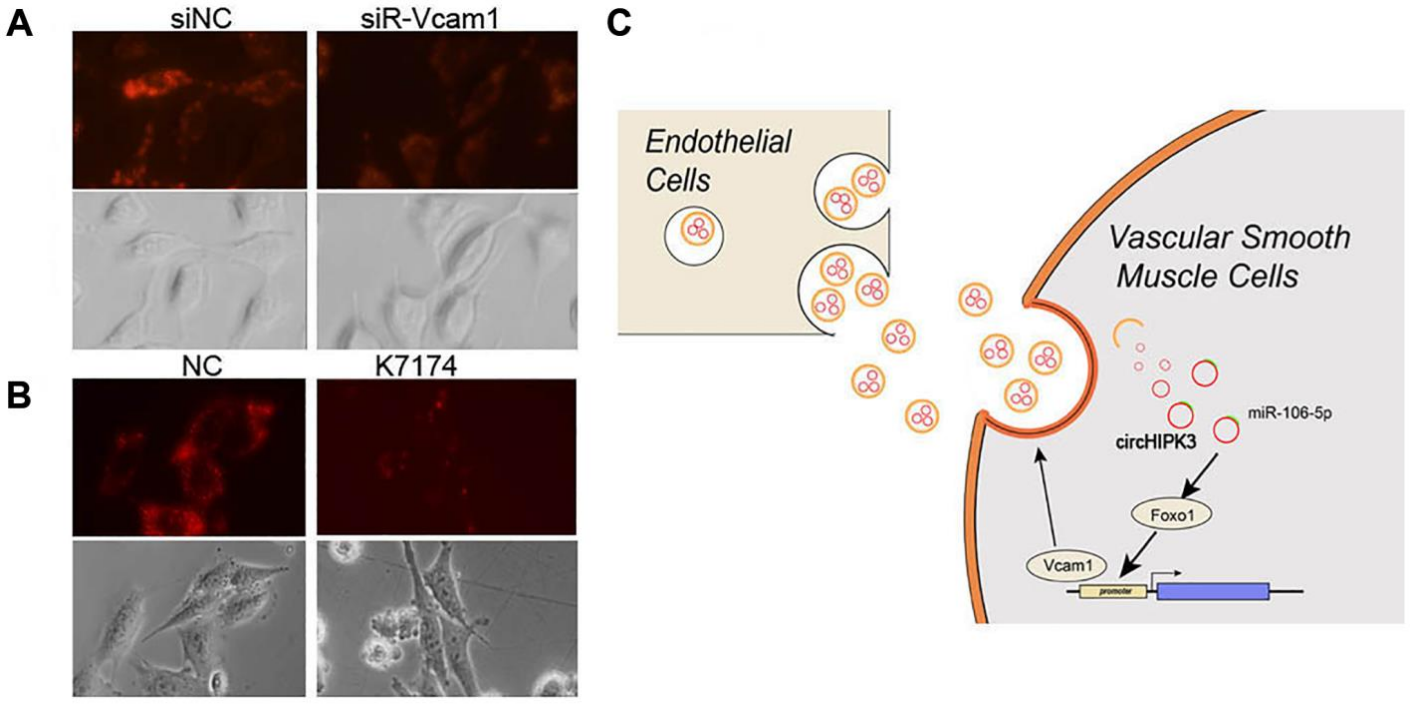
**Vcam1 mediated the EC uptake of EC-derived exosomes in VSMCs.** (**A**–**B**) DiI-labeled exosomes derived from MAECs exposed to VSMCs when Vcam1 was (**A**) knocked down or (**B**) treated with K-7174. (**C**) The overview of this research.

Exosome uptake partly occurs via adhesion factors, including Vcam1, which induces fusion and endocytosis. Subsequently, we aimed to study whether EC-derived exosome uptake was depended on Vcam1-mediated endocytosis. We used DiO-labeled exosomes to analyze the uptake of exosomes, which decreased when Vcam1 expression was knocked down by siRNA (Figure 8A). K-7174, which is a Vcam1 inhibitor, could also block the uptake of exosomes (Figure 8B). These results indicated that the uptake of EC-derived exosomal circHIPK3 by VSMCs partly depends on Vcam1-mediated endocytosis.

## DISCUSSION

Recent studies have revealed that the intake of HG is a major determinant of vascular complications in diabetes, which can lead to increased inflammation and endothelial dysfunction [15]. The progress of vascular damage is related to multiple steps and results from the interaction between various types of cells. However, the crosstalk and downstream changes between ECs and VSMCs are largely unknown. The main findings related to the present study are as follows: i) Exosomal circHIPK3 excreted by MAECs promoted the proliferation and inhibited the apoptosis of VSMCs. ii) Exosomal circHIPK3 sponged miR-106a-5p to relieve the repression of Foxo1 expression. iii) Foxo1, which is a transcription factor, promoted Vcam1 expression, which facilitated the uptake of MAEC-derived exosomes by VSMCs (Figure 8C).

ECs and VSMCs are the two major cells in the blood vessels. The crosstalk between them is essential not only in the development and maintenance of the function of blood vessels but also in the pathologic process of vascular complications. The communication between ECs and VSMCs could take place in indirect (biochemical) ways [16]. Werner [17] showed that ECs could secrete microparticles that carry miR-126-3p to inform the proliferation of VSMCs. In the present study, we demonstrate that in an HG condition, MAECs promote the proliferation and inhibit the apoptosis of VSMCs. Specifically, these effects are partly rescued by the addition of GW4869, which is an exosome inhibitor. These effects lead us to consider the influences of EC-derived exosomes on VSMC proliferation. Exosomes are a subset of extracellular vesicles with a diameter of 40–150 nm [3]. We isolated exosomes from culture medium in an NG or HG concentration and found that the median was 59.25 nm for NG-Exo and 69.25 nm for HG-Exo detected by NTA. Exosome surface markers (CD63 and TSG101) were positively expressed in these vesicles, thus further verifying the existence of exosomes in the culture medium. These EC-derived exosomes also promote the proliferation and inhibit the apoptosis of VSMCs.

Exosomes exhibit their functions depending on their nucleic acids, proteins, and lipids [18]. Ling et al. [19] found that 217 circRNAs were upregulated and 484 ciRNAs were downregulated in HG treated glomerular ECs. We also found high levels of circHIPK3 in MAEC-derived exosomes in an HG condition. circHIPK3 is located on chromosome 11p13, which is involved in medicating a wide range of physiological and pathological processes of cardiovascular disease [20]. HG can upregulate circHIPK3 expression in retinal EC. Knocking down circHIPK3 expression blocks the progression of vascular dysfunction [13]. In the present study, we found that circHIPK3 derived from MAECs promoted the proliferation and inhibited the apoptosis of VSMCs. We also examined the effect of circHIPK3 on the VSMC contraction induced by acetylcholine. Knocking down of circHIPK3 expression promoted the acetylcholine-induced VSMC contraction.

circRNAs act as the molecular sponge of miRNA to relieve the repression of mRNA expression. For example, circHIPK3 sponges miR-93-5p to promote Rac expression, which aggravates infarction-induced cardiac dysfunction [21]. Another study found that HG-induced circHIPK3 sponges miR-29b to abolish the inhibition of AKT3 expression by miR-29b, which protects cardiomyocytes against ischemia/reperfusion injury [22]. We also attempt to construct the circRNA–miRNA–mRNA network in VSMCs. We perform bioinformatics to predict the potential miRNAs sponged by circHIPK3 from GEO dataset (GSE73058). A total of 28 miRNAs were predicted to have binding sites with circHIPK3. Dual luciferase assay was used to filter the target miRNAs binding to circHIPK3 and showed that miR-106a-5p have binding sites with circHIPK3. Their regulatory relationship was confirmed by dual luciferase assay and qRT-PCR. Nevertheless, bioinformatics also predicted that miR-106a-5p could target Foxo1 3′UTR and negatively regulate its expression. The regulatory relationship between miR-106a-5p and Foxo1 was verified by qRT-PCR, western blot analysis, and IF. Thus, we confirm that circHIPK3 sponges miR-106a-5p to relieve the repression of Foxo1 expression. In the functions of this regulatory axis, miR-106a-5p decreases proliferation and increases apoptosis in VSMCs. The roles of miR-106a-5p in proliferation and apoptosis is consistent with the circHIPK3/miR-106a-5p/Foxo1 axis in VSMCs.

Foxo1 is a key regulator of glucose homeostasis and insulin sensitivity. Under an HG condition, Foxo1 transcriptional activity is related to diabetic complications [23]. The elevation of Foxo1 expression is a promoter of diabetic vascular remodeling [24]. Our results are consistent with perspectives stating that Foxo1 promotes the proliferation and inhibits the apoptosis of VSMCs. As a transcription factor, Foxo1 is a positive regulator of Vcam1 [25]. We also verified that Foxo1 promotes Vcam1 expression, which further promotes VSMC proliferation. Vcam1, which is also known as CD106, is a cell adhesion molecule involved in reticulocyte-derived exosomes binding to fibronectin. Anti-Vcam1 inhibits Tspan8-induced exosome uptake in ECs. The enrichment of Vcam1 on the surface of exosomes enhances the uptake of exosomes by ECs [26]. We found a reduction of exosome uptake in VSMCs when Vcam1 was knocked down by siRNA or its inhibitor, thus indicating that exosome uptake by VSMCs is partly performed by Vcam1. Our results exhibit a novel regulatory mechanism in which exosomal circHIPK3 secreted by MAECs sponges miR-106a-5p to relieve its repression of Foxo1, thus facilitating the uptake of MAEC-derived exosomes by VSMCs.

## MATERIALS AND METHODS

### Cell culture and transfection

In this experiment, MAECs (Cat.CL0200) were purchased from Fenghbio (China). Mouse VSMCs and 293A cells were donated by the Key Laboratory of Neural and Vascular Biology. All cells were cultured in Dulbecco’s Modified Eagle Medium (DMEM; Hyclone, UT) containing 10% fetal bovine serum (FBS; Excellbio, Cat. FSD500, China) and 1% penicillin/streptomycin (PS; Solarbio, Cat. P1400, China) in a humidified incubator at 37°C with 5% CO_2_. D-glucose (Sigma, MO) was used to generate hyperglycemic conditions after the cells were cultured in DMEM medium containing 2% FBS and 1% PS for 24 h. For cell co-culture, MAECs were pre-cultured on the transwell overnight, and the MAEC-transwell was loaded into another 24-well plate that was cultured with VSMCs for 48 h. For exosome-related cell culture, cells were cultured in DMEM medium containing 2% FBS (Excellbio, China) without exosomes by ultracentrifugation and 1% PS. For cell transfection, Lipofectamine 2000 (Invitrogen, CA) was used to transfect cells according to the manufacturer’s protocol. Briefly, 1 μg plasmid or 20 pmol RNA was mixed in Opti-MEM™ (Thermo Fisher, MA) in room temperature for 15 min. In this mixture, cells were incubated for 4 h, and we replaced the DMEM medium after the incubation. For luciferase reporter assay, 0.5 μg plasmid and 10 pmol RNA were mixed together and co-transfected with 1 μl Lipofectamine 2000.

### Isolation and identification of exosomes

We collected an MAEC medium (50 ml) that was treated in 5.6 or 30 mM glucose for 72 h and was centrifuged at 2000 × g for 30 min at 4°C. Thereafter, the supernatant was collected for further experiments. This supernatant was filtered through a 0.22 μm filter membrane after centrifugation at 10,000 × g in 4°C for 45 min (CP100MX, Hitachi, Japan). The supernatant was ultracentrifuged at 100,000 × g in 4°C for 70 min. The supernatant was then discarded. We resuspended the pellet with 5 ml PBS and subjected it to ultracentrifugation at 100,000 × g in 4°C for 70 min. The pellet was then collected, resuspended in 100 μL PBS, and stored at −80°C.

### TEM and NTA

A 20 μL exosome suspension was pipetted out on a carbon-film copper mesh for 5 min. Filter paper was used to absorb the excess liquid, and 2% phosphotungstic acid (Sinotech Genomics, China) was dropped on a carbon-film copper mesh for 1–2 min. The excess liquid was then absorbed and dried at room temperature. A transmission electron microscope (HT-7700, Hitachi, Japan) was used to observe and collect the image. The size and concentration of exosomes was analyzed with NTA (N30E, NanoFCM, China).

### Exosome labeling and cellular uptake

Exosomes were labeled with 1,1’ dioctadecyl l–3,3,3’, 3’-tetramethylindocarbocyanine perchlorate (DiO; Sigma, MO) according to the manufacturer’s instructions. DiI was added in PBS containing exosomes for 20 min and was washed twice at 100,000 × g for 70 min to remove free DiI dye. After resuspension in PBS, DiI-labeled exosomes were added into a medium at a final concentration of 3 μg/mL at 37°C for 6 h. 4’, 6-Diamidino-2-phenylindole (DAPI; Sigma, MO) was added for 10 min and imaged by a fluorescence microscope (IX73, Olympus, Japan).

### Plasmid construction and RNA interference

Recombinant plasmids or mutation plasmids were synthesized by Genecreate, China. We designed siRNAs against circHIPK3, Foxo1, and Vcam1, and the siRNAs were synthesized by Genecreate, China. Table 1 shows the target sequences of siRNA.

**Table 1:** Primers and RNA sequences used in this study.

circHIPK3 F	GGATCGGCCAGTCATGTATC
circHIPK3 R	ACCGCTTGGCTCTACTTTGA
miR-106a-3p RT	CTCAACTGGTGTCGTGGAGTCGGCAATTCAGTTGAGCTACCT
miR-106a-3p F	ACACTCCAGCTGGGCAAAGTGCTAACAG
U6 F	CTCGCTTCGGCAGCACA
U6 R	AACGCTTCACGAATTTGCGT
Reverse	TGGTGTCGTGGAGTCG
Foxo1 F	AATTCGGTCATGCCAGCGTA
Foxo1 R	AAGCGGTTCATGGCAGATGT
Vcam1 F	CTGGGAAGCTGGAACGAAGT
Vcam1 R	GCCAAACACTTGACCGTGAC
Gapdh F	GTCAAGGCTGAGAACGGGAA
Gapdh R	AAATGAGCCCCAGCCTTCTC
miR-106a-5p mimics	CAAAGTGCTAACAGTGCAGGTAG
miR-106a-5p inhibitor	CTACCTGCACTGTTAGCACTTTG
SiR-circHIPK3	GGCCAUACCUGUAGUAGCGAG
SiR-Foxo1	GAAAGAGTTCTTGGTGGATGCTCAA
SiR-Vcam1	TGGCTCCAGACATTTACCCAGTTTA

### RNA isolation and RT-qPCR

Total RNA was extracted using TRIzol (Solarbio, China) according to the manufacturer’s protocol. After washing cells with PBS, 1 ml TriQuick Reagent was used to cover the cultured cells, and the cells were lysed completely. Thereafter, 200 μL chloroform was added, shaken vigorously, and left at room temperature for 2 min. It was then centrifuged at 12000 g for 10 min in 4°C, and the upper phase was transferred to another tube. A total of 0.5 ml isopropanol was added, mixed, and settled at room temperature for 10 min following centrifugation at 12,000 × g for 10 min in 4°C. Subsequently, 75% ethanol was used to wash the sediment after centrifugation. DNase and RNase free water was used to dissolve the RNA pellet. A total of 1 μg of RNA was added to 1 μL specific miR-106-5p or U6 RT primers at 65°C for 10 min, at 25°C for 5 min, and in ice for 2 min. The strand buffer, M-MLV, and dNTPs (Beyotime, China) were added to the mixture at 42°C for 30 min and at 70°C for 10 min, and the mixture was stored at −20°C. For long mRNA, we used a random primer to perform reverse-transcription PCR at 65°C for 10 min and in ice for 2 min, followed by 42°C for 30 min and 70°C for 10 min; it was then stored at −20°C. In real-time PCR, a 2 μL reverse transcription product was used to perform amplification according to the manufacturer’s protocol (Servicebio, China). Amplification was conducted at 95°C for 3 min, followed by 40 cycles at 95°C for 12 sec and 62°C for 40 sec. A relative level of transcripts was normalized by the 2^−ΔΔCq^ method. Gapdh or U6 were used to normalize the level of large mRNAs or miRNAs. Table 1 lists the specific primers used.

### Dual luciferase reporter assay

293A cells were co-transfected by pmirGLO reporter plasmids (wild type or mutant) and miRNA mimics. 293A cells were seeded into a 24-well plate and then transfected. The cells were lysed by RIPA (Solarbio, China) after 48 h. Luciferase activity was measured using a Dual-Glo Luciferase Assay System (Promega, WI) with a Flash and Glow reader. The relative luciferase activity was calculated by the ratio of firefly luciferase to Renilla luciferase.

### CCK-8

VSMCs were seeded in a 96-well plate with a density of 3000 cells per well. After incubating in 5% CO_2_ atmosphere at 37°C for 24 h, 10 μL of CCK8 (Solarbio, China) solution was added to the medium. Three repetitions were made during the experiments. The optical density (OD) values were measured at 450 nm by using a microplate reader (MD2, Molecular Devices, CA).

### EdU assay

The DNA synthesis of VSMCs was performed using the BeyoClick™ EdU Cell Proliferation Kit with Alexa Fluor 488 (Beyotime, China) according to the manufacturer’s instructions. After transfection, EdU working solution was added into the medium containing VSMCs for 2 h. The cells were washed with PBS and fixed in 4% paraformaldehyde. Thereafter, a permeabilization solution was used to incubate cells at room temperature for 15 min, and a reaction solution was used to incubate cells in the dark at room temperature for 30 min. Flow cytometry (BD FACSCanto II, BD Biosciences, NJ) was used to detect the fluorescence intensity.

### FCM for apoptosis

Cells were cultured in a six-well plate after transfection. Annexin V-FITC/PI apoptosis kit (Liankebio, China) was used according to the manufacturer’s instructions for the detection of cell apoptosis. A total of 1 × 10^5^ cells were collected and stained with FITC and PI in a binding buffer. Cell apoptosis was detected under BD FACS Canto II (BD Biosciences, NJ).

### Cell contraction assay

The contractility of VSMCs was detected by using the Confocal Laser Scanning Microscope System (Leica, GER). The cells were stimulated with 100 nM acetylcholine (Ach) for 30 s. Before and after the stimulation by Ach, the length of cells was analyzed by Image-Pro Express. The relative length of cells was calculated as the ratio of cells length to siR-NC group before Ach stimulation.

### Animal models

All animals were housed and cared for according to the guidelines and regulations of the Institutional Animal Care and Use Committee of Hebei Medical University. The db/db mice are a model of diabetes mellitus type 2 with hyperglycemia, while db/m mice served as the control. The mice were housed at 12:12 h light-dark room in Key Laboratory of Neural and Vascular Biology. The aortas of db/db mice and db/m mice were collected and fixed in 4% paraformaldehyde.

### IF

Cells and tissues were fixed in 4% paraformaldehyde and incubated in 0.3% Triton 100 for 10 min. Anti-Foxo1 (AF6416, Affbiotech, China) and anti-Vcam1 (DF6082, Affbiotech, China) were used to incubate cells overnight. Secondary antibody (5230–0332, KPL, IN) was used to incubated cells for 1 h the next day after washing with PBS. DAPI (Solarbio, China) was used to stain the nucleus. DNA synthesis was imaged by a fluorescence microscope (IX73, Olympus, Japan).

### Western blot analysis

Isolated exosomes or cells were homogenized in RIPA buffer (Solarbio, China). Protein concentration was measured by a BCA protein assay kit (Beyotime, China). The lysates of cells, exosomes, or cells were separated by SDS-PAGE and then transferred to Polyvinylidene fluoride (PVDF) membranes (Millipore, MA). These PVDF membranes were blocked for 2 h and incubated in primary antibody TSG101 (67381-1-lg, Proteintech, China), CD63 (67605-1-lg, Proteintech, China), Vcam1 (DF6082, Affbiotech, China), Foxo1 (AF6416, Affbiotech, China), Bax (GTX109683, Genetex, CA), Bcl2 (RT1069, Huabio, China), Caspase 3 (1087-1, Epitomics, China), and PCNA (610664, BD, NJ) overnight. Secondary antibodies (5230-0336 and 5230-0341, KPL, IN) were incubated for 1 h in room temperature. The obtained bands were explored using an enhanced chemiluminescence reagent and were imaged by ChemiDoc MP (Bio-Rad, CA). β-actin was used as an internal control.

### Statistical analysis

All statistical analyses were performed using GraphPad Prism (version 5.0; GraphPad Software, Inc. CA). Obtained data were shown as means ± standard deviation. Student’s *t*-test was employed to compare the statistical differences between two groups. All experiments were performed in triplicate independently. A value of *p* ≤ 0.05 was considered statistically significant.
